# A High-fat and High-Cholesterol Diet Potentiates Oxidative Damage in Hippocampus of Mice Lacking Apolipoprotein E

**DOI:** 10.2174/1874205X01812010012

**Published:** 2018-02-21

**Authors:** Mary Guaraldi, Thomas B. Shea

**Affiliations:** Laboratory for Neuroscience, Department of Biological Sciences, University of Massachusetts Lowell, Lowell, MA 01854, USA

**Keywords:** Oxidative damage, Latent oxidative damage, ApoE, Hippocampus

## Abstract

**Objective:**

We examined genetic and dietary challenge on hippocampal oxidative damage. Mice expressing (ApoE+/+) or lacking apolipoprotein E (ApoE-/-) were maintained on a standard diet or a high fat /high cholesterol (challenge) diet for 11-31 weeks. Similar levels of oxidative species were observed for ApoE+/+ and -/- mice maintained on the basal diet.

**Method:**

However, treatment of ApoE-/- homogenates with hydrogen peroxide and iron increased oxidative species by >100%, indicating an equivalent amount of potential oxidative species in latent form. We observed a time- and region-specific induction of oxidative damage in the hippocampi of ApoE-/- but not +/+ mice while maintained on the challenge diet. Notably, however, additional significant latent oxidative products were detected during this time. After 31 weeks of dietary challenge, by which time hippocampal oxidative species had doubled, there was an additional 50% in the latent form.

**Conclusion:**

This highlights the degree to which ApoE deficiency places hippocampal tissue at risk for oxidative damage. Even a modest dietary deficiency may be sufficient to provoke oxidative damage to hippocampal tissue. These results highlight the combinatorial impact of genetic and dietary deficiencies on oxidative damage to hippocampal tissue.

## 1 INTRODUCTION

In addition to their well-known deleterious impact on cardiovascular physiology [1], diets high in saturated fat have been implicated in contributing to cognitive decline and neuronal degeneration observed with aging and with diseases such as Alzheimer’s Disease (AD) [2-8].

Cholesterol and its metabolism may also play a role in the pathogenesis of AD. The incidence of AD is higher in countries with high-fat, high-cholesterol diets [9]. Cholesterol-lowering drugs may reduce the manifestation of AD in persons at risk [10]. High-fat diets can lead to increases in free radical formation [11] and oxidative stress is a major contributing factor to AD [12, 13]. Endogenous antioxidants such as glutathione help to prevent oxidative damage to tissues [14]. Dietary supplementation with folic acid, vitamin E, and other antioxidants has been shown to slowdown the progress of oxidative stress in brain tissue with subsequent reduction in cognitive deficits in mouse models [11, 14].

Apolipoprotein E (ApoE) is involved in lipid delivery and metabolism and is important for growth and repair of the nervous system. ApoE synthesis increases after neuronal injury and in AD [2, 14, 15] and may influence the degree of oxidative stress-induced damage in the frontal cortex of AD patients [16]. The E4 allele of ApoE has been implicated as a genetic risk for AD [17]. Transgenic mice lacking murine ApoE (ApoE-/- mice) demonstrated increased susceptibility to oxidative damage than their wild-type counterparts, which can be accompanied by neurodegenerative alterations and cognitive deficiencies, due at least in part to impaired antioxidant activity [2, 16-20]. These mice therefore, represent a useful model for genetic predisposition to oxidative damage.

Herein, we examined the influence of a diet high in saturated fat and cholesterol on oxidative damage in hippocampal tissue of ApoE+/+ mice and ApoE-/- mice. We focused on the hippocampus due to its central and critical role in memory and since it displays some of the earliest impacts on AD [21, 22].

## 2 MATERIALS and METHODS

### 2.1 Mice and Treatments

C57BL/6 mice homozygously expressing (ApoE+/+; Taconic Farms, Germantown, NY; n = 12) or lacking apolipoprotein E (ApoE-/-; bred in-house from stock originating from Jackson Labs, Bar Harbor, ME; n = 39) were maintained from weaning on a standard (“Basal”) diet (catalog #5755, Test Diets, Richmond, IN) consisting of 21% vitamin-free casein, 15% sucrose, 43.65% Dextrin, 5% corn oil, 5% lard, 3% non-nutritive fiber, RP vitamin and mineral mixtures, DL-methionine and choline chloride. This diet provided 19% protein, 10% fat, 4.3% crude fiber, 60.6% carbohydrate and 4.08kcal/g gross energy. The mice were then maintained for up to 31 weeks on a “high fat/high cholesterol (“Challenge”) diet (catalog #21551; Test Diets) consisting of 31.6% sucrose, 21% milk fat, 19.5% casein, 10% maltodextrin, RP vitamin and mineral mixtures, DL methionine, choline chloride, and 0.15% cholesterol (with ethoxyquin as a preservative). This diet provided 16.8% protein, 20.3% fat, 6.5% fiber, 48.8% carbohydrate, 2056ppm cholesterol and 4.52kcal/g gross energy.

### 2.2 Tissue preparation

The mice were euthanatized by CO_2_ asphyxiation followed by decapitation in accord with Institutional Care and Use Committee approval. Brains were rapidly removed and stored at -80^o^C until assayed. Hippocampi were weighed and homogenized with 9 volumes of ice-cold double-distilled H_2_O containing 0.4% butylated hydroxytoluene to inhibit auto-oxidation. Homogenates (50μg total protein) in 5mM HEPES containing 1μM copper sulfate were sonicated on ice (2 x 3sec) then incubated for 60min at 37^o^C. Additional samples also received 0.1mM iron sulfate (JT Baker, Phillipsburg, NJ) and 0.1mM hydrogen peroxide (EMD Chemicals, Gibbstown, NJ) to maximize direct production of oxidation products [23].

### 2.3 Measurement of oxidation products

Thiobarbituric acid reactive substances (TBARS) were quantified as described [25] with minor modifications as follows: 1ml of 15% trichloroacetic acid containing 0.375% TBA and 0.25N HCl was added to the incubation mixture. Samples were vortexed, incubated at 90^o^C for 30 min, placed on ice for 4min, followed by the addition of 4mL butanol; pyridine (15:1). Samples were vigorously shaken and then centrifuged (1500 x g, 10min at 4^o^C). The supernatant was decanted and assayed in a Shimadzu model 1501 spectrofluorophotometer (Shimadzu Scientific Instruments, Columbia, MD) with excitation and emission wavelengths at 520nm and 553nm, respectively. The assay was repeated 3 – 4 times for each experimental group and µM TBARS/mg total protein was determined by comparison to a standard curve of 1,1,3,3-tetramethoxypropane in 0.1N HCl.

Values are presented as mean ± standard error of the mean (SEM) for 6 – 12 mice per group. Statistical analyses were carried out for all conditions via ANOVA with post-hoc comparisions via Fisher’s LSD and 2-tailed Student’s *t* tests. Reagents were obtained from Sigma-Aldrich (St Louis, MO) unless otherwise specified.

## 3 RESULTS

**Fig. (1):**
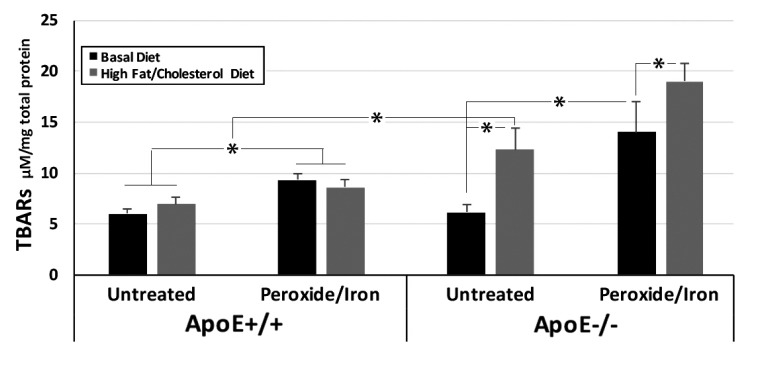
Impact of the Challenge diet on oxidative species in hippocampi of mice expressing or lacking ApoE.

ApoE+/+ and -/- mice displayed identical levels of oxidative species in hippocampi when maintained on a standard diet. We converted all latent oxidative products to TBARS by the treatment of hippocampal homogenates with the pro-oxidants hydrogen peroxide plus iron. The conversion of latent oxidative species to TBARs revealed significant latent oxidative species in hippocampi of ApoE-/- mice maintained on a standard diet. By contrast, no latent products were detected in hippocampi of ApoE+/+ mice (Fig. **1**).

Maintenance on the Challenge diet for 31 weeks induced an approximately 50% increase in TBARS in hippocampal homogenates from ApoE+/+mice. An identical increase in TBARs was observed following the treatment of these homogenates with peroxide and iron, indicating that no additional latent oxidative products accumulated within ApoE+/+ hippocampi in response to this dietary challenge.

A 200% increase in oxidative species was observed in hippocampi of ApoE-/- mice following maintenance on the Challenge diet for 31 weeks. Treatment of these homogenates with peroxide and iron induced a further 48% increase in TBARs, indicating the presence of significant latent oxidative products. As in prior studies on dietary deficiency [24], maintenance of ApoE-/- mice on the challenge diet impaired Y maze navigation by 10% (55±3% at baseline; 49±6% after 31 weeks on the Challenge diet), consistent with a deleterious impact on hippocampal function.

Values represent µM TBARs / mg total protein (mean ± standard error of the mean) in hippocampal homogenates from mice on the Basal diet or after 31 weeks on the Challenge diet, prior to and following treatment with peroxide and iron (n = 6 ApoE+/+ mice for each time point; n = 9 ApoE-/- mice for Basal and 12 for High fat/high cholesterol diets). Overall values among group were statistically different (*p* <0.001; ANOVA) *p<0.05 in post-hoc comparisons via *t* test.

**Fig. (2):**
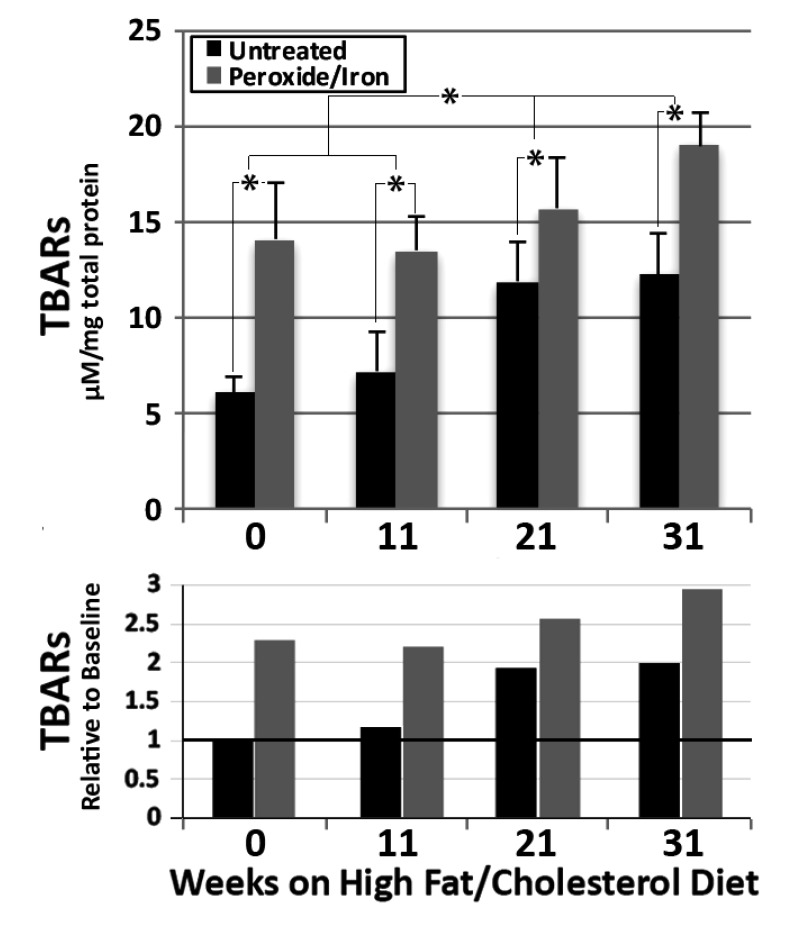
Progressive increase in oxidative species following maintenance of ApoE-/- mice on the Challenge diet.

We monitored the time course of the increase in TBARS in ApoE-/- hippocampal homogenates. TBARs progressively increased over 21 weeks of maintenance on the high fat/high cholesterol diet, with no further increase by 31 weeks (Fig. **2**). Treatment with peroxide and iron significantly increased TBARs at all times, indicating the presence of significant levels of latent oxidative products. A progressive significant increase in these latent oxidative products was observed over 31 weeks (Fig. **2**).

The upper graph presents µM TBARs / mg total protein (mean ± standard error of the mean) in hippocampal homogenates from ApoE-/- mice maintained for 0-31 weeks on the Challenge diet, prior to and following treatment with peroxide and iron (n = 9 for 0, 11 and 21 weeks and 12 for 31 weeks High fat/high cholesterol diets)). Overall values among group were statistically different (*p* <0.01; ANOVA) *p<0.05 in post-hoc comparisons via *t* test.

The lower graph presents values for all times and conditions relative to time 0 (i.e., basal diet) without treatment with peroxide and iron, where this basal value is defined as 1.

## 4 DISCUSSION

ApoE-/- mice are in general more vulnerable to oxidative stress, excitotoxic injury and ischemia [25-27], due, at least in part, to their inherently impaired antioxidant mechanisms [17-21, 28]. Oxidative damage in hippocampus is consistent with our previous demonstration of cognitive decline following maintenance of ApoE-/- mice on a diet that fostered oxidative damage to overall brain tissue, and alleviation of cognitive decline by supplementation with antioxidants [15]. In prior studies, we examined the impact of dietary challenge on cortical tissue of ApoE-/- mice [20, 21]. Herein, we focused on hippocampal tissue since hippocampal function is critical in memory consolidation and displays some of the earliest signs of degeneration in AD [22, 23, 29]. An increase in oxidative species was to be anticipated [15, 26, 30] but what is particularly novel is the observation of a tremendous “reserve” of potential oxidative damage in the form of latent oxidative products. Even a modest dietary deficiency may therefore, be sufficient to provoke oxidative damage to hippocampal tissue.

It has been difficult to demonstrate unequivocal impact of diet on dementia [8, 30]. Notably, unlike pharmacological studies, nutritional studies are inherently compromised due to the difficulty of recruiting sufficient numbers of individuals who have inadequate baseline nutrition, such that a cohort with defined initiation of supplementation can be compared to a placebo cohort not receiving supplementation. While nutritional intervention may more likely to be effective for individuals who had prior chronic insufficiency; that very population is more likely to already have latent damage that may preclude any beneficial effect. However, animal studies, which are readily controlled, support these hypotheses. For example, maintenance on a high-fat diet induced a decline in synaptic transmission, synapsin, learning and memory, brain-derived neurotrophic factor, cognitive performance and increased brain lesions and cognitive problems in rodent model [11, 29, 31-35]. These deleterious impacts can manifest during juvenile years [36], and in some instances, as a consequence of maternal consumption [31].

These findings add to a growing body of evidence that key dietary deficiencies can contribute to the onset of AD in some cases, by exacerbating otherwise latent genetic risks including the increased propensity for oxidative damage inherent in ApoE deficiency [30].
